# TBCRC 057: Survey about willingness to participate in cancer clinical trials during the pandemic

**DOI:** 10.1002/cam4.7090

**Published:** 2024-03-11

**Authors:** Karen Lisa Smith, Carolyn Mead‐Harvey, Gina L. Mazza, Eileen H. Shinn, Elizabeth S. Frank, Michelle E. Melisko, Cyd Eaton, Yisi Liu, Jeannine M. Salamone, Teri Pollastro, Patricia A. Spears, Nicole E. Caston, Antonio C. Wolff, Gabrielle Betty Rocque

**Affiliations:** ^1^ Sidney Kimmel Comprehensive Cancer Center, Women's Malignancies Disease Group Johns Hopkins University School of Medicine Baltimore Maryland USA; ^2^ Department of Quantitative Health Sciences Mayo Clinic Scottsdale Arizona USA; ^3^ Department of Behavioral Science, Division of Cancer Prevention and Population Sciences The University of Texas MD Anderson Cancer Center Houston Texas USA; ^4^ Dana‐Farber Cancer Institute Boston Massachusetts USA; ^5^ Department of Medicine (Hematology/Oncology) University of California San Francisco California USA; ^6^ Biostatistics, Epidemiology and Data Management Core Johns Hopkins University School of Medicine Baltimore Maryland USA; ^7^ Georgetown Breast Cancer Advocates Washington District of Columbia USA; ^8^ University of Washington Seattle Washington USA; ^9^ Lineberger Comprehensive Cancer Center University of North Carolina at Chapel Hill Chapel Hill North Carolina USA; ^10^ Division of Hematology and Oncology University of Alabama at Birmingham (UAB) Birmingham Alabama USA; ^11^ Division of Gerontology, Geriatrics, and Palliative Care University of Alabama at Birmingham (UAB) Birmingham Alabama USA; ^12^ Present address: AstraZeneca Gaithersburg Maryland USA

**Keywords:** breast cancer, clinical trials, COVID‐19, pandemic, survey

## Abstract

**Background:**

Breast cancer patients experienced heightened anxiety during the pandemic. Also, modifications to clinical trial activities allowing for virtual platforms, local assessments, and greater flexibility were introduced to facilitate participation. We sought to evaluate the association between pandemic‐related anxiety and willingness to participate in trials and how pandemic‐era modifications to trial activities affect the decision to participate.

**Methods:**

We conducted an online survey from August to September, 2021 of patients with breast cancer assessing pandemic‐related anxiety; clinical trials knowledge and attitudes; willingness to participate during and before the pandemic; and how each modification affects the decision to participate. Fisher's exact tests evaluated differences in proportions and two‐sample *t*‐tests evaluated differences in means. The association of pandemic‐related anxiety with a decline in willingness to participate during compared to prior to the pandemic was modeled using logistic regression.

**Results:**

Among 385 respondents who completed the survey, 81% reported moderate–severe pandemic‐related anxiety. Mean willingness to participate in a trial was lower during the pandemic than prior [2.97 (SD 1.17) vs. 3.10 (SD 1.09), (*p* < 0.001)]. Severe anxiety was associated with higher odds of diminished willingness to participate during the pandemic compared to prior (OR 5.07). Each of the modifications, with the exception of opting out of research‐only blood tests, were endorsed by >50% of respondents as strategies that would increase their likelihood of deciding to participate.

**Conclusions:**

While pandemic‐related anxiety was associated with diminished willingness to participate in trials, the leading reasons for reluctance to consider trial participation were unrelated to the pandemic but included worries about not getting the best treatment, side effects, and delaying care. Patients view trial modifications favorably, supporting continuation of these modifications, as endorsed by the National Cancer Institute and others.

## 1 INTRODUCTION

Clinical trials have led to advances in treatment for breast cancer that have improved survival.^1^, ^2^ Guidelines endorse participation in a clinical trial as the preferred treatment for patients with breast cancer whenever possible.^3^ However, fewer than 10% of adult cancer patients enroll in a clinical trial.^4^, ^5^ Longstanding recognized barriers to participation include narrow access based on strict eligibility criteria; the geographic concentration of trials at academic centers; stakeholders' limited knowledge about trials and negative attitudes towards trials; among others.^4^, ^5^, ^6^, ^7^, ^8^, ^9^, ^10^, ^11^, ^12^, ^13^, ^14^ The burden of trial participation placed on patients has also increasingly been recognized as a barrier. Trial activities often require participants to take time away from family and work, bearing associated costs for travel, lodging, lost work hours, and childcare.^6^, ^7^, ^12^ Consideration of participant burden is a key target for interventions to increase trial enrollment.

Since early 2020, the COVID‐19 pandemic has disrupted oncology care, including clinical trial operations, with many aspects of research‐related and routine care being delayed, transitioned to virtual platforms, or even omitted.^15^, ^16^, ^17^, ^18^, ^19^, ^20^ In the setting of pandemic‐related restrictions regarding trial enrollment, site closures, delayed opening of new trials, and limitations in investigational drug availability, trial accrual sharply declined in the first few months of the pandemic.^15^, ^17^, ^20^, ^21^, ^22^, ^23^ Accrual rates have gradually recovered since then, with smaller declines coinciding with upticks in COVID‐19 case numbers.^22^, ^24^

Acknowledging the importance of patient safety and the operational challenges associated with clinical research during the pandemic, research and regulatory agencies issued guidelines supporting modification of trial activities.^25^, ^26^ These guidelines allowed for delay or omission of the non‐essential activities and for the completion of some activities at local sites or using virtual platforms. In general, these modifications were patient‐centric, enhancing convenience, and reducing the burden of participation. As such, continuation of many of these modifications, both during the pandemic and afterwards, has been widely endorsed,^18^, ^20^, ^27^, ^28^, ^29^, ^30^, ^31^, ^32^, ^33^, ^34^, ^35^, ^36^, ^37^, ^38^, ^39^, ^40^ and may provide opportunities for long‐term increases in trial participation.

Anxiety levels among patients with breast cancer, which were already elevated compared to the general population prior to the pandemic, have increased during the pandemic.^41^, ^42^, ^43^, ^44^, ^45^ Among cancer survivors who reported in Spring 2020 that the pandemic made them less likely to participate in trials, the fear of increased exposure to COVID‐19 was a leading reason cited for reduced likelihood of participation.^46^ The relationship between pandemic‐related anxiety and willingness to participate in clinical trials as the pandemic continues in the era of available COVID‐19 vaccines is unknown. Thus, the Translational Breast Cancer Research Consortium (TBCRC) conducted a survey of patients with breast cancer to evaluate the effect of the pandemic on willingness to participate in trials. We hypothesized that higher pandemic‐related anxiety would be associated with a decline in willingness, even in a cohort with access to COVID‐19 vaccines. We also hypothesized that pandemic‐era modifications to trial activities would make patients more likely to decide to participate in a trial.

## 2 METHODS

### 2.1 Study design and recruitment strategy

We conducted a cross‐sectional online survey in a convenience sample of patients with breast cancer. Respondents were primarily recruited through social media. Promotional material and the survey link were shared with breast cancer patient advocacy organizations who promoted the survey through their online platforms. TBCRC investigators were also encouraged to promote the survey through their social media networks. The survey link could be forwarded to allow for snowball recruitment.^47^ Printed recruitment materials were also available in breast cancer clinics at Johns Hopkins. The survey link was open for 8 weeks. Initiation of the online survey served as informed consent to participate. This research was approved by the Johns Hopkins Institutional Review Board.

The primary objective was to describe breast cancer patients' willingness to participate in trials during the pandemic compared to prior to the pandemic and to evaluate associations between pandemic‐related anxiety and receipt of COVID‐19 vaccination with willingness to participate. Key secondary objectives were to (a) determine whether pandemic‐era modifications to trial activities would influence the decision to participate, (b) describe attitudes towards clinical trials during the pandemic and, (c) to describe reasons for reluctance to participate in trials during the pandemic.

### 2.2 Eligibility criteria

Eligible respondents were English‐ or Spanish‐speaking US residents aged ≥18 years who met one or both of the following criteria: (1) Self‐reported diagnosis of breast cancer of any stage within 5 years or (2) Self‐reported diagnosis of metastatic breast cancer (MBC) at any time. The first five survey questions determined eligibility. Only eligible respondents were allowed to complete the remainder of the survey.

### 2.3 Survey

The survey was developed by a multi‐disciplinary TBCRC team including medical oncologists, behavioral scientists, and patient advocates. After clicking the survey link, respondents completed the survey in English or Spanish on the Johns Hopkins University REDCap platform.^48^, ^49^ The survey did not collect health information identifiers.

Respondents self‐reported demographics, breast cancer characteristics, and breast cancer treatment including location of care and use of telemedicine. Exposure to SARS‐CoV‐2 and perceived prior COVID‐19 infection were assessed using modified versions of questions from the All of Us COVID‐19 Participant Experience (COPE) Survey.^50^ Respondents reported COVID‐19 vaccination status using questions modified from the Patient Advocate Foundation longitudinal COVID‐19 patient survey.^51^ Those who were unvaccinated indicated vaccine intent and reason(s) for lack of intent. Prior COVID‐19 testing and results thereof were assessed using questions modified from Center of Disease Control & Prevention/National Institutes of Health Common Data Element Bank items.

Anxiety about the pandemic was assessed using an 11‐point numerical analog scale (NAS) ranging from “0, no anxiety” to “10, worst anxiety possible”. Prior literature demonstrates correlation between scores on an 11‐point NAS assessing fear/anxiety of SARS‐CoV‐2 and a validated coronavirus‐specific anxiety measure.^45^, ^52^

Knowledge about clinical trials was assessed with 11 true/false items, seven of which were created by Ellis et al. for a study assessing the relationship between clinical trials knowledge and willingness to participate in trials.^53^ Four additional questions were created for this survey.

Attitudes towards clinical trials were assessed with the Attitudes Towards Cancer Trials Scales–Cancer Treatment Subscale (ACTS‐CT). This 18‐question measure includes four domains that reflect attitudes towards clinical trials: Personal Beliefs (four items), Personal Barriers/Safety (five items), Personal and Social Value (five items), and Trust in the Research Process (four items). Responses are reported using a 7‐point scale from “1, strongly disagree” to “7, strongly agree”. Negatively worded items are reverse‐coded such that higher scores indicate better attitudes towards trials.^54^ We modified the ACTS‐CT by substituting the word “cancer study” with “clinical trial” in order to maintain consistent terminology throughout our survey. We also included three additional questions about attitudes towards trials developed by Melisko et al to address concerns related to delays in care, time off work, and out‐of‐pocket costs associated with clinical trial participation.^7^ We modified the response scale for these three questions to a 7‐point scale to match the ACTS‐CT response options and reverse‐coded negatively worded items such that higher scores indicated better attitudes towards trials for these three questions also.

Respondents were asked whether they had discussed clinical trials with a provider and whether they had participated in a trial, indicating if these events occurred prior to or during the pandemic. To establish pre‐pandemic baseline willingness, respondents were asked to rate their willingness to participate in a trial prior to the pandemic on a 5‐point scale from “0, not at all willing” to “4, definitely willing”. Using the same scale, respondents were asked to indicate their willingness to participate during the pandemic. Respondents who were participating in a trial during either time period were considered “definitely willing” for that time period. Respondents who were not current trial participants and who indicated anything other than being “definitely willing” to participate during the pandemic were considered reluctant to participate during the pandemic and asked their reasons for reluctance.

The effect of pandemic‐era modifications to clinical trial activities on a respondent's decision to participate in a trial during or after the pandemic was assessed with 11 questions grouped into categories related to change in location of the trial activity from the trial site to closer to home (blood tests, imaging, and use of local providers for toxicity assessments), use of virtual platforms for trial activities (provider telemedicine visits, online consent, and online study questionnaires), and adding flexibility/convenience (limited frequency of study visits, choice to opt out of research‐only blood tests and biopsies, widening windows for study activities, and home delivery of oral study medications). Respondents rated how each modification would affect the decision to participate on a 5‐point scale ranging from “1, much less likely to participate” to “5, much more likely to participate”.

### 2.4 Statistical analysis

Results are presented descriptively using means [standard deviation (SD)], medians (interquartile range), frequencies, proportions, and box‐and‐whisker plots. For categorical variables, differences in proportions between groups were evaluated using Fisher's exact tests. For continuous variables, mean differences between groups were evaluated using equal variance two‐sample *t*‐tests. Mean difference in willingness to participate in a breast cancer clinical trial before versus during the pandemic was evaluated using a paired *t*‐test.

Correct responses to the knowledge assessment items were summed to derive the Knowledge Score (range 0–11), with higher scores indicating greater knowledge. Responses to the ACTS‐CT items were summed to derive the subdomain scores (range 5–35 for 5‐item domains and 4–28 for 4‐item domains) and the Global ACTS‐CT Score (range 18–126) with higher scores indicating better attitudes. Change in willingness to participate in a trial during the pandemic compared to prior was dichotomized (decline in willingness versus no decline in willingness). Pandemic‐related anxiety was categorized as none/mild (0–3), moderate (4–6) or severe (7–10). The association of pandemic‐related anxiety and other factors with a decline in willingness was modeled with univariate and multivariable logistic regression. Variables significant on univariate analysis were selected for the multivariable model.

Since the survey was open for a fixed time period, pre‐determination of the sample size was not possible. Thus, analyses are not powered for hypothesis testing and are exploratory without adjustment for multiplicity. Two‐sided tests were used and *p*‐values <0.05 were considered significant. Analyses were conducted using SAS 9.4 (SAS Institute Inc., Cary, NC) and R 4.1.2.

## 3 RESULTS

### 3.1 Respondent characteristics

**FIGURE 1:**
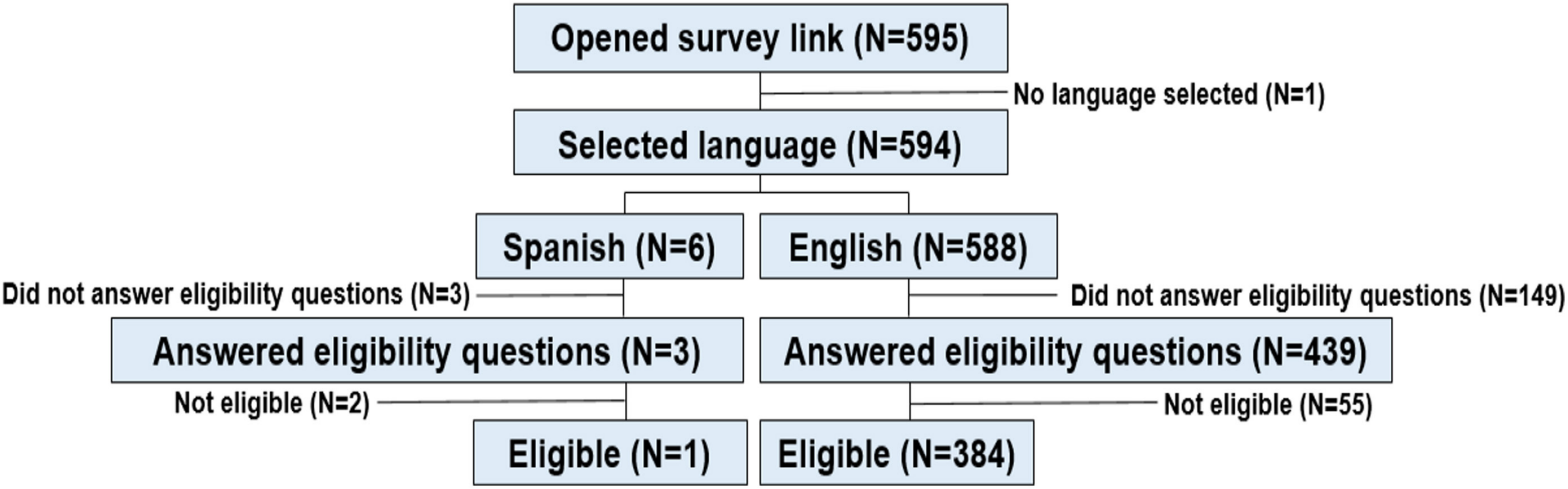
Consort diagram. Figure displays the numbers of respondents who opened the survey link, selected their preferred language for completing the survey (English or Spanish), answered the eligibility questions and who were ultimately eligible to participate.

The survey was open August 6, 2021–September 30, 2021, during which time 595 respondents opened the link, 385 of whom were eligible and completed the survey. (Figure 1). Most respondents were White (73%), had more than high school education (80%) and lived in urban areas (71%) in neighborhoods with poverty rates <15% (64%). Forty percent received care at an academic center and 72% traveled <60 min to their providers. Approximately half had MBC and 89% were actively receiving breast cancer therapy. Most (88%) were vaccinated against COVID‐19 (Table 1).

**TABLE 1:** Respondent characteristics.

Characteristic	*N* = 385
*Demographics*	
Median age in years (range)	52 (25–85)
Gender—*N* (%)	
Cisgender female	278 (72.2)
Cisgender male	1 (0.3)
Chose not to disclose or missing	106 (27.5)
Race—*N* (%)	
White/Caucasian	279 (72.5)
Black/African American	10 (2.6)
Other race/more than one race	18 (4.7)
Missing	78 (20.3)
Ethnicity—*N* (%)	
Hispanic	14 (3.6)
Non‐Hispanic	302 (78.4)
Missing	69 (17.9)
Highest level of academic achievement—*N* (%)	
Less than high school diploma or equivalent	1 (0.3)
High school diploma or equivalent	7 (1.8)
Some college, no degree	43 (11.2)
Associate degree	20 (5.2)
Bachelor degree	122 (31.7)
Master or doctoral degree	123 (31.9)
Missing	69 (17.9)
Marital status—*N* (%)	
Single/widowed/divorced/separated	74 (19.2)
Married/partnered	237 (61.6)
Other	4 (1.0)
Missing	70 (18.2)
Live with school‐age children—*N* (%)	
No	218 (56.6)
Yes	97 (25.2)
Missing	70 (18.2)
Employment status—*N* (%)	
Work full time (≥32 h/week)	121 (31.4)
Work part time (<32 h/week)	39 (10.1)
Unemployed/not working/retired/on disability/other	151 (39.2)
Missing	74 (19.2)
Household income—*N* (%)	
≤$49,999	54 (14.0)
$50,000–$99,999	86 (22.3)
>$100,000	165 (42.9)
Missing	80 (20.8)
Neighborhood poverty rate^a^—*N* (%)	
<15%	248 (64.4)
≥15%	64 (16.6)
Missing	73 (19.0)
Location of residence^b^—*N* (%)	
Rural (RUCA 4–10)	40 (10.4)
Urban (RUCA 1–3)	272 (70.6)
Missing	73 (19.0)
Health insurance^c^—*N* (%)	
Private	238 (61.8)
Medicare	77 (20.0)
Medicaid	13 (3.4)
Tricare/other military health insurance	10 (2.6)
Veteran's health administration	4 (1.0)
Other health insurance	10 (2.6)
No health insurance	1 (0.3)
Missing	74 (19.0)
*Breast cancer disease and treatment characteristics*	
Extent of disease—*N* (%)	
Early stage	183 (47.5)
Metastatic	202 (52.5)
Years since breast cancer diagnosis—*N* (%)	
<1 year	84 (21.8)
1–5 years ago	178 (46.2)
5–10 years ago	66 (17.1)
>10 years ago	57 (14.8)
Age at breast cancer diagnosis—*N* (%)	
<50 years	191 (49.6)
≥50 years	125 (32.5)
Missing	69 (17.9)
Primary location of breast cancer care—*N* (%)	
Academic medical center	154 (40.0)
Community based/private practice	136 (35.3)
Not sure	27 (7.0)
Missing	68 (17.7)
Travel time to breast cancer provider—*N* (%)	
<30 min	198 (51.4)
30–60 min	80 (20.8)
>60 min	39 (10.1)
Missing	68 (17.7)
Actively receiving treatment^d^—*N* (%)	
Yes	344 (89.4)
No	26 (6.8)
Missing	15 (3.9)
*Experience during COVID‐19 pandemic*	
COVID‐19 community transmission level^e^—*N* (%)	
High (≥100 cases/100,000)	272 (70.6)
Substantial (50–99.99 cases/100,000)	20 (5.2)
Moderate (10–49.99 cases/100,000)	15 (3.9)
Low (0–9.99 cases/100,000)	5 (1.3)
Missing	73 (19.0)
Ever been near someone with known or suspected COVID‐19—*N* (%)	
Yes	124 (32.2)
No	261 (67.8)
Ever been tested for COVID‐19—*N* (%)	
Yes	317 (82.3)
No	57 (14.8)
Missing	11 (2.9)
Ever tested positive for COVID‐19—*N* (%)	
Yes	30 (7.8)
No	286 (73.3)
Missing	69 (17.9)
Think you ever had COVID‐19—*N* (%)	
Yes/maybe	66 (17.1)
No	307 (79.7)
Missing	12 (3.1)
Received ≥1 dose of COVID‐19 vaccine—*N* (%)	
Yes	339 (88.1)
No^f^	30 (7.8)
Missing	16 (4.1)

Abbreviations: RUCA, Rural–Urban Commuting Area Codes; SES, socioeconomic status.

^a^ Neighborhood poverty rate, the percentage of individuals residing in a ZIP code whose family income is below the federal poverty level, was determined from US Census data based on ZIP code. Neighborhood poverty rate ≥15% is considered a surrogate for low SES.

^b^ Urban versus rural residence was determined using Rural–Urban Commuting Area Codes based on ZIP code.

^c^ Respondents were allowed to select >1 type of insurance.

^d^ Respondents were considered to be actively receiving treatment if they indicated that they had undergone surgery, received radiation or received chemotherapy for breast cancer within the past 6 weeks or that they were taking oral medication for breast cancer.

^e^ COVID‐19 community transmission level in the 7 days leading up to the midpoint of the time period the survey was open (i.e., in the 7 days leading up to September 3, 2021) is presented. COVID‐19 community transmission level was determined based on county using the Centers for Disease Control and Prevention COVID tracker at https://covid.cdc.gov/covid‐data‐tracker/#datatracker‐home.

^f^ Among the 30 unvaccinated respondents, 2 (6.7%), 1 (3.3%), 9 (30%), 4 (13.3%), and 14 (46.7%) indicated they were extremely likely, somewhat likely, unsure, somewhat unlikely and extremely unlikely to get the vaccine when available to them, respectively. Among those who were unsure, somewhat unlikely or extremely unlikely, the primary reasons were concerns re vaccine safety (69.2%), health condition may limit ability to get vaccine (7.7%), and other reason (23.1%).

### 3.2 Pandemic‐related anxiety, clinical trials knowledge, clinical trials attitudes and clinical trials experience

The mean (SD) pandemic‐related anxiety score was 5.5 (2.31). 161 (43%), and 142 (38%) respondents reported moderate and severe pandemic‐related anxiety, respectively. (Figure S1, online only). The mean (SD) Knowledge Score was 7.9 (3.03). The proportions of respondents whose responses were correct for each knowledge assessment item ranged from 44% to 89% (Table S1, online only). The mean (SD) Global ACTS‐CT Score was 92 (13.74) (Table 2). Eighty‐eight (23%) respondents reported discussing a trial with a provider during the pandemic and 37 (10%) respondents were current trial participants (Table S2, online only).

**TABLE 2:** Attitudes towards clinical trials.

	Mean (SD)
ACTS‐CT^a^	
Global ACTS‐CT score	92 (13.74)
Personal beliefs subdomain score	15.1 (5.17)
Personal barriers/safety subdomain score	22.8 (5.95)
Personal and social value subdomain score	31 (4.29)
Trust in the research process subdomain score	23 (4.30)
Additional attitudes items^b^	
1. I will get my needed treatment as soon as possible if I am in a clinical trial	4.6 (1.49)
2. If I am in a clinical trial I will have to spend extra time having more tests and doctor visits	2.7 (1.26)
3. I may have to spend more time and money on transportation and childcare and may lose income due to time away from work if I participate in a clinical trial	3.7 (1.76)

Abbreviations: ACTS‐CT, Attitudes Towards Cancer Trials Scales–Cancer Treatment Subscale; SD, standard deviation.

^a^ ACTS‐CT items were scored using a 7‐point Likert scale ranging from “1, strongly disagree” to “7, strongly agree.” Negatively worded items were reverse coded. Responses were summed to derive the global and sub‐domain scores. Higher scores indicate better attitudes.

^b^ Mean scores for additional attitudes items are reported individually (range 1–7). Additional items #2 and #3 were reverse coded. Higher scores indicate better attitudes towards trials.

### 3.3 Willingness to participate in a clinical trial

**FIGURE 2:**
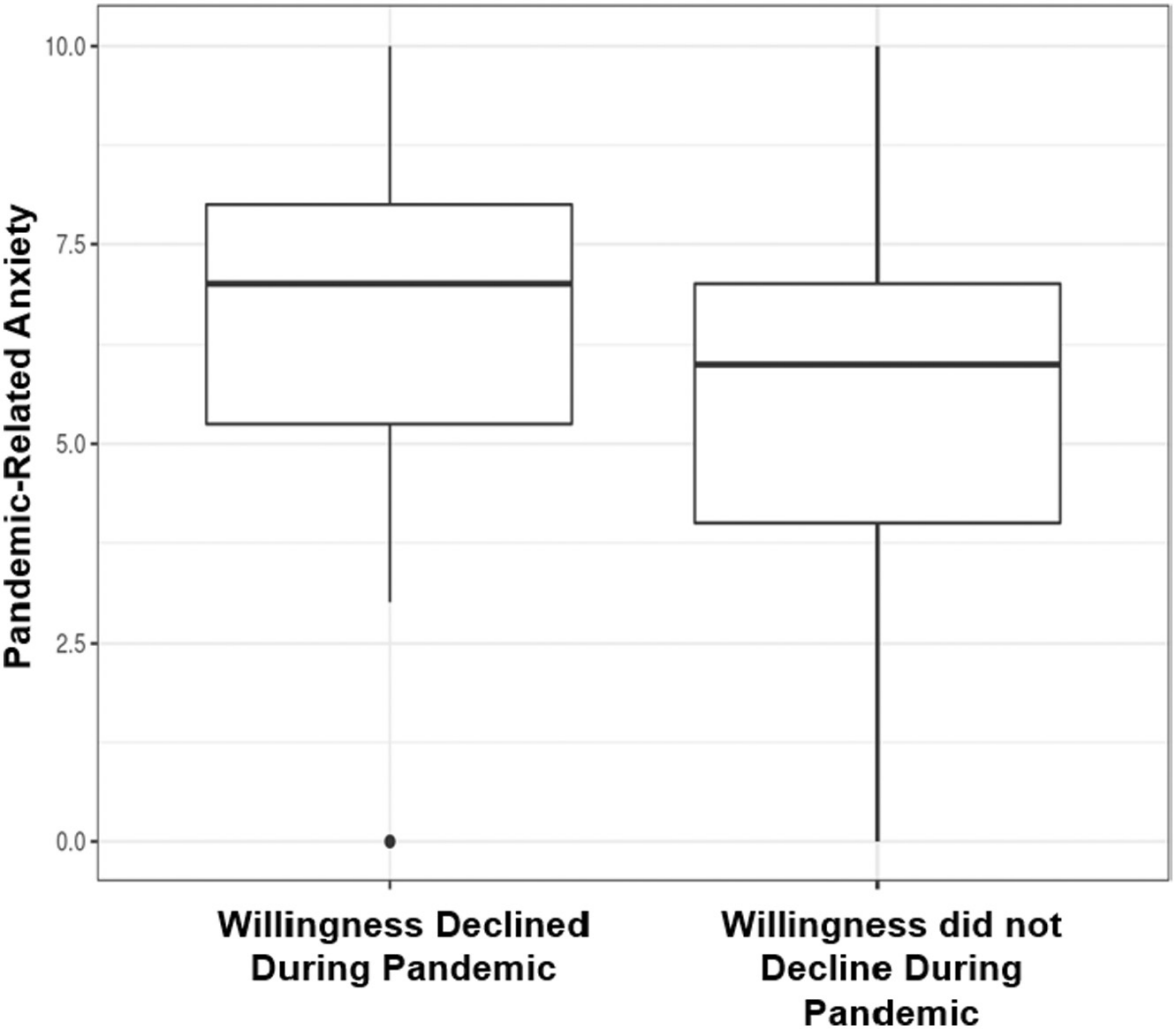
Pandemic‐related anxiety according to whether or not willingness to participate in a trial declined during the pandemic. Figure displays box‐and‐whisker plots of pandemic‐related anxiety scores for respondents whose willingness to participate in a trial during the pandemic compared to prior to the pandemic declined (left) and for respondents whose willingness to participate in a trial during the pandemic compared to prior did not decline (right). Pandemic‐related anxiety was rated on an 11‐point scale (0‐no anxiety to 10‐worst anxiety possible). Willingness to participate in a clinical trial was assessed on a 5‐point scale from “0, not at all willing” to “4, definitely willing”. Respondents indicated their willingness to participate before and during the pandemic separately. Respondents diagnosed during the pandemic were asked to consider their willingness prior to the pandemic under the hypothetical condition that their diagnosis occurred prior to the pandemic. Prior trial participants were considered to have been “definitely willing” to participate before the pandemic and current trial participants were considered “definitely willing” to participate during the pandemic. Current trial participants whose participation began prior to the pandemic were considered to be “definitely willing” to participate both before and during the pandemic. Change in willingness to participate in a trial was calculated by subtracting the willingness to participate score before the pandemic from that during the pandemic, with negative values indicating a decline in willingness to participate in a trial during the pandemic compared to prior. Change in willingness was categorized as binary variable—decline in willingness versus no decline in willingness.

**FIGURE 3:**
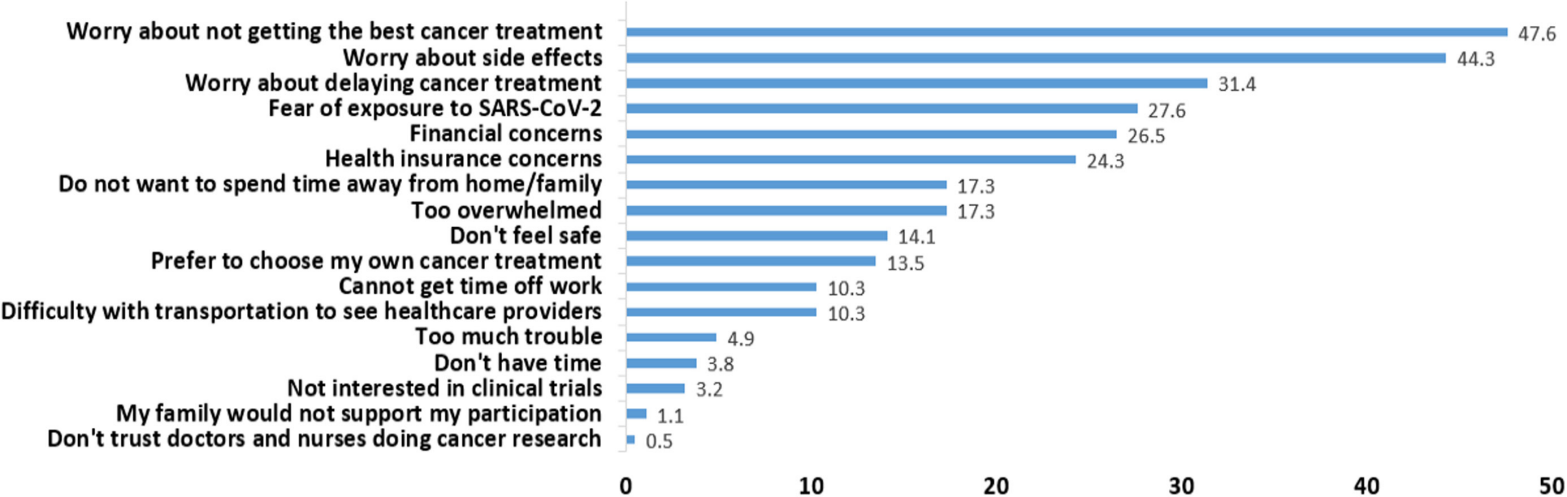
Reasons for reluctance to participate in clinical trials during the pandemic. Figure displays the percentage of the 185 respondents who were reluctant to participate in a clinical trial during the pandemic who selected each reason for their reluctance. Respondents could select >1 reason. Respondents who were not current trial participants and who indicated anything other than being “definitely willing” to participate during the pandemic were considered reluctant to participate during the pandemic.

Mean willingness to participate in a trial was lower during the pandemic than prior (2.97 versus 3.10, *p* < 0.001). Fifty (13%) respondents had a decline in willingness to participate during compared to prior to the pandemic. Mean pandemic‐related anxiety was 6.6 among respondents with a decline in willingness compared to 5.5 among respondents without a decline (*p*‐value 0.002; Figure 2). On multivariable modeling, individuals with severe anxiety had 5.07 times the odds of having a decline in willingness. After controlling for covariates, for every 1‐point increase in Global ACTS‐CT Score and every 1‐point increase in Knowledge Score, the odds of having a decline in willingness decreased by 3% and 15%, respectively. Univariate regression did not identify an association between COVID‐19 vaccination and a decline in willingness (Table 3). Almost half (48%) of the respondents were reluctant to participate in a trial during the pandemic, 51 (28%) of whom cited fear of exposure to SARS‐CoV‐2 as a reason for their reluctance (Figure 3).

**TABLE 3:** Univariate and multivariable logistic regression modeling of factors associated with a decline in willingness to participate in trials during compared to prior to the pandemic.

Variable		OR (95% CI)	*p*‐value
*Univariate logistic regression*			
Extent of disease	Metastatic versus early stage	1.21 (0.66–2.22)	0.54^a^
Primary location of breast cancer care	Academic medical center versus other	0.87 (0.46–1.62)	0.65^a^
Age in years	<50 versus ≥50	1.31 (0.72–2.38)	0.38^a^
Race/ethnicity	Other versus non‐Hispanic White	1.87 (0.79–4.42)	0.16^a^
Prior or current trial participation	Yes versus No	1.00 (0.47–2.10)	0.99^a^
Prior discussion of a trial with a provider	Yes versus No	0.55 (0.29–1.04)	0.66^a^
Received ≥1 dose of COVID‐19 vaccine	Yes versus No	1.41 (0.41–4.85)	0.59^a^
Highest level of academic achievement			0.73^b^
High school diploma (or equivalent) or less versus master or doctoral degree		1.94 (0.36–10.40)	0.44^a^
Some college (no degree) or associate degree versus master or doctoral degree		0.73 (0.29–1.85)	0.51^a^
Bachelor degree versus master or doctoral degree		1.02 (0.50–2.07)	0.96^a^
Pandemic‐related anxiety^c^			0.04^b^
Moderate versus none/mild		3.00 (0.86–10.50)	0.09^a^
Severe versus none/mild		4.66 (1.36–15.99)	0.01^a^
Global ACTS‐CT Score	1 point increase	0.96 (0.94–0.99)	0.001^a^
Knowledge Score	1 point increase	0.84 (0.75–0.95)	0.004^a^
*Multivariable logistic regression* ^d^			
Pandemic‐related anxiety			0.02^b^
Moderate versus none/mild		2.64 (0.71–9.8)	0.15^a^
Severe versus none/mild		5.07 (1.39–18.47)	0.01^a^
Global ACTS‐CT Score	1 point increase	0.97 (0.95–0.99)	0.01^a^
Knowledge Score	1 point increase	0.85 (0.75–0.98)	0.02^a^

Abbreviations: ACTS‐CT, Attitudes Towards Cancer Trials Scales–Cancer Treatment Subscale; CI, 95% confidence interval; OR, odds ratio.

^a^ Covariate Wald *p*‐value.

^b^ Type 3 Wald *p*‐value.

^c^ Pandemic‐related anxiety was rated on an 11‐point scale (0‐no anxiety to 10‐worst anxiety possible). Scores of 0–3 were considered none/mild, scores of 4–6 were considered moderate and scores of 7–10 were considered severe.

^d^ Variables significant on univariate analysis were selected for the multivariable model.

### 3.4 Effect of pandemic‐era modifications to clinical trial activities on decision to participate in a trial

**FIGURE 4:**
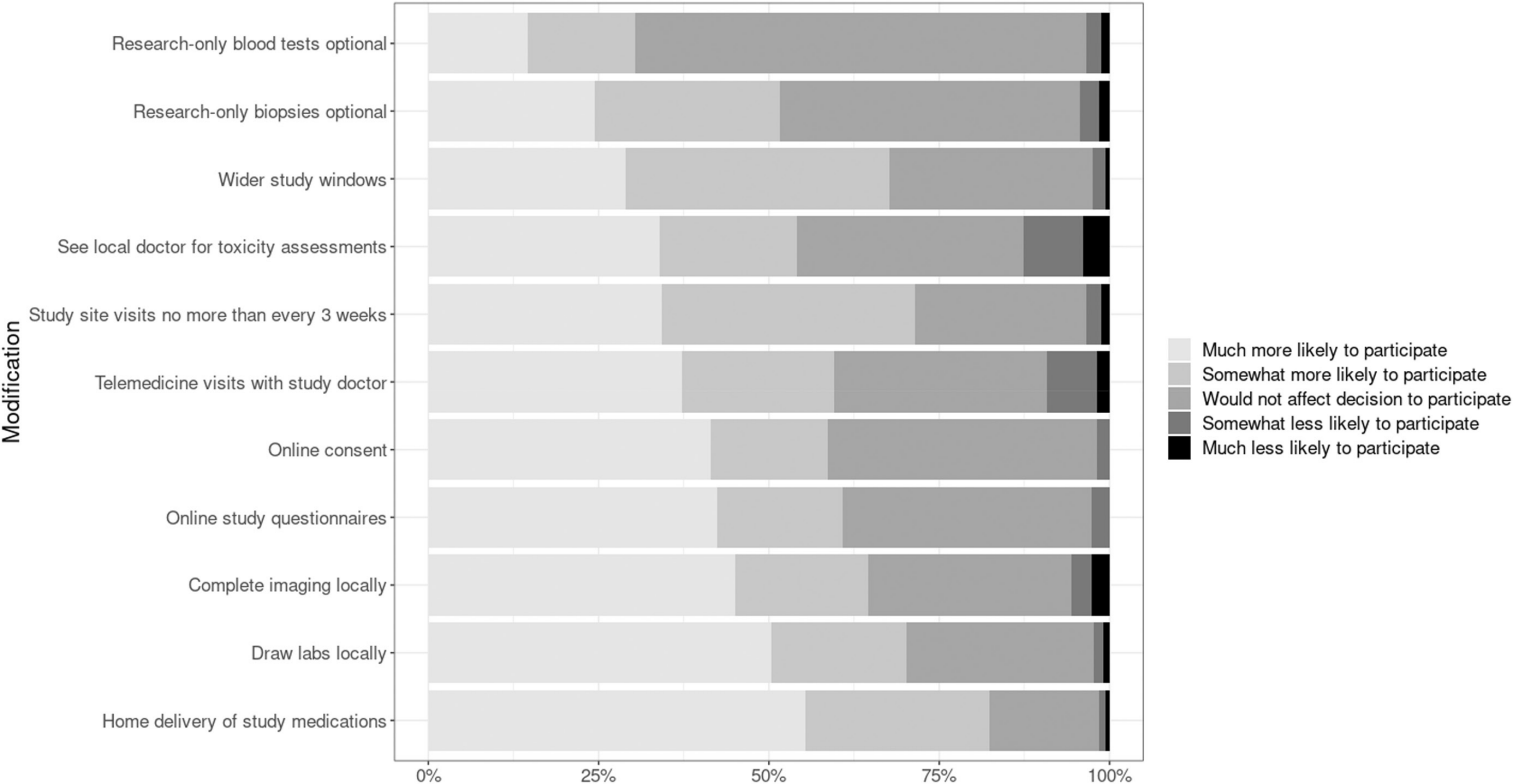
The effect of pandemic‐era modifications to clinical trial activities on decision to participate in a trial during or after the pandemic. For each modification to clinical trial activities, the figure displays the proportions of respondents who indicated they would be much less likely to participate (orange), somewhat less likely to participate (khaki), would not affect the decision whether or not to participate (green), somewhat more likely to participate (blue) and much more likely to participate (pink) in a trial during or after the pandemic. Current trial participants were asked to consider how modifications would affect their decision to participate in a future trial. Percentages <15% are not displayed.

With the exception of allowing research‐only blood tests to be optional, >50% of respondents indicated that each of the following modifications to clinical trial activities would make them somewhat or much more likely to decide to participate in a trial: changing the location of trial activities from the trial site to closer to home (blood tests, imaging, and use of local providers for toxicity assessments); the use of virtual platforms for trial activities (provider telemedicine visits, online consent, and online study questionnaires); and adding flexibility/convenience (allowing research‐only biopsies to be optional, study visits no more frequent than once every 3 weeks, widening windows for study activities, and home delivery of oral study medications; Figure 4). Respondents who cited fears of exposure to SARS‐CoV‐2 as a reason for reluctance to participate in trials during the pandemic viewed the modifications related to use of virtual platforms more favorably than did respondents who did not cite fear of exposure to SARS‐CoV‐2 as a reason for their reluctance to participate during the pandemic (Table S3, online only).

## 4 DISCUSSION

In this cross‐sectional online survey of patients with breast cancer conducted 18 months after the onset of the COVID‐19 pandemic, we observed ongoing high rates of pandemic‐related anxiety despite the availability of COVID‐19 vaccines. Almost half the respondents were reluctant to participate in a trial well after the pandemic began. Moderate or severe pandemic‐related anxiety was associated with 5‐fold higher odds of having a decline in willingness to participate in a trial during the pandemic compared to prior. Our survey is one of the first to directly report the association between anxiety and willingness to participate in a clinical trial during the pandemic in patients with breast cancer.

In our survey, 13% of respondents had a decline in willingness to participate in a trial during the pandemic compared to prior, a proportion fairly similar to that reported by Fleury et al in a survey of cancer survivors of all types conducted May–June 2020. Notably, the leading reason for being less likely to participate in a trial in Fleury et al's survey was fear of exposure to SARS‐CoV‐2 (70% of respondents), a reason cited by only 28% of our survey respondents who were reluctant to consider trial participation during the pandemic.^46^ Indeed, the leading reasons for reluctance to consider trial participation reported in our study were unrelated to the pandemic–worries about not getting the best treatment, side effects, and delaying care. This difference in reasons for reluctance to participate between our survey and Fleury et al's survey may be attributable to differences in respondent populations, growing acceptance of COVID‐19 as part of a “new normal,” or the availability of COVID‐19 vaccines since Fleury et al's survey.

Many researchers have endorsed the pandemic‐era modifications to trial activities, suggesting that the pandemic has provided an opportunity to forever change how clinical trials are designed and conducted, with an eye towards increasing efficiency and patient‐centeredness.^18^, ^20^, ^27^, ^28^, ^29^, ^30^, ^31^, ^32^, ^33^, ^34^, ^35^, ^36^, ^37^, ^38^, ^39^, ^40^ Our survey takes this a step further, demonstrating that most pandemic‐era modifications to trial activities (change in location from the trial site to closer to home, use of virtual platforms, and adding flexibility/convenience) are viewed favorably by patients with breast cancer and that these modifications may affect the decision to participate in a trial. Pandemic‐era modifications to trial activities align with new National Cancer Institute led efforts to streamline clinical trials that include goals such as reducing participant burden, performing trial procedures locally or via virtual platforms, and increasing access and accrual to trials.^55^, ^56^

It is not known whether patients with cancer are aware of the pandemic‐era modifications to clinical trial activities. In our survey, greater knowledge about and better attitudes towards clinical trials were associated with lower odds of having a decline in willingness to participate Educating patients about the pandemic‐era modifications to trial activities that reduce the burden of participation may improve knowledge about and attitudes towards clinical trials, thereby enhancing willingness to participate.

Our findings align with those reported by de Las Heras et al in a survey addressing comfort with de‐centralized clinical trials among patients with cancer, defined as trials in which aspects of care are provided at home instead of at the trial site. In their survey, mean comfort levels on a scale from 1 to 5 exceeded 4 for home delivery of study medication, telemedicine visits, home visits for study assessments, online questionnaires, and wearing mobile devices.^57^ Together with our survey, Las Heras et al's findings support ongoing implementation of pandemic‐era modifications that allow for trial activities to be completed away from the study site whenever possible.

Limitations of our study include that it may not be generalizable to all patients with cancer as respondents were limited to patients with breast cancer with internet access and most were non‐Hispanic White, well educated, urban residents, of high socioeconomic status, vaccinated against COVID‐19, and ultimately, these patient populations are typically those who are offered clinical trials by their physicians. Future studies in more diverse populations, including those who have been historically under‐represented in clinical trials are needed.^5^ Although receipt of COVID‐19 vaccination did not mitigate the odds of having a decline in willingness to participate in a trial during the pandemic compared to prior in our study, our ability to assess the relationship between vaccination and a willingness to participate was limited as our cohort was predominantly vaccinated. Furthermore, our definition of reluctance to participate in a trial (which included all respondents who were non‐participants and who answered anything other than definitely willing to participate) was selected in order to maximize collection of information about reasons for unwillingness to participate; had we selected a different definition, our findings may have differed. Another limitation of our study is that we did not collect information on the types of cancer treatments (oral versus intravenous) received by the survey participants, and the type of treatment and associated side effects may have influenced the survey. In addition, respondent fatigue led to incomplete data, particularly on demographic questions at the end of the survey. Finally, our analyses were not powered for hypothesis testing and we did not adjust for multiplicity.

In conclusion, we demonstrated that pandemic‐related anxiety is associated with the diminished willingness of patients with breast cancer to participate in clinical trials in an era where vaccines were available. However, pandemic‐era modifications to clinical trial activities positively influenced the decision to participate, suggesting that rethinking trial activities to be more patient‐centric and to decrease the burden of participation by continuing pandemic‐era modifications is an important strategy to enhance trial participation moving forward.

## AUTHOR CONTRIBUTIONS

**Karen Lisa Smith:** Conceptualization (equal); data curation (equal); formal analysis (equal); funding acquisition (lead); writing – original draft (equal); writing – review and editing (equal). **Carolyn Mead‐Harvey:** Formal analysis (equal); writing – original draft (equal); writing – review and editing (equal). **Gina L. Mazza:** Formal analysis (equal); writing – original draft (equal); writing – review and editing (equal). **Eileen H. Shinn:** Conceptualization (equal); data curation (equal); formal analysis (equal); writing – original draft (equal); writing – review and editing (equal). **Elizabeth Frank:** Conceptualization (equal); data curation (equal); formal analysis (equal); writing – original draft (equal); writing – review and editing (equal). **Michelle Melisko:** Conceptualization (equal); data curation (equal); formal analysis (equal); writing – original draft (equal); writing – review and editing (equal). **Cyd Eaton:** Data curation (equal); project administration (equal); writing – original draft (equal); writing – review and editing (equal). **Yisi Liu:** Writing – original draft (equal); writing – review and editing (equal). **Jeannine M. Salamone:** Conceptualization (equal); data curation (equal); formal analysis (equal); writing – original draft (equal); writing – review and editing (equal). **Teri Pollastro:** Conceptualization (equal); data curation (equal); formal analysis (equal); writing – original draft (equal); writing – review and editing (equal). **Patricia A. Spears:** Conceptualization (equal); data curation (equal); formal analysis (equal); writing – original draft (equal); writing – review and editing (equal). **Nicole E. Caston:** Formal analysis (equal); writing – original draft (equal); writing – review and editing (equal). **Antonio Wolff:** Conceptualization (equal); data curation (equal); formal analysis (equal); writing – original draft (equal); writing – review and editing (equal). **Gabrielle Betty Rocque:** Conceptualization (equal); data curation (equal); formal analysis (equal); writing – original draft (equal); writing – review and editing (equal).

## FUNDING INFORMATION

This work was conducted by the Translational Breast Cancer Research Consortium and supported by the Metastatic Breast Cancer Network.

## CONFLICT OF INTEREST STATEMENT

Dr. Smith receiving research grants to institution from Pfizer, spouse has stock in Abbvie and Abbott Labs, and is currently employed at AstraZeneca. Dr. Melisko receives research funding from OBI Pharma, Daehwa, and Novartis. Dr. Rocque received research funding from Genentech, Pfizer, and Carevive and consulting fees for Genentech and Pfizer.

## PRIOR PRESENTATIONS

Portions of this work were presented as a poster at the American Society of Clinical Oncology Annual Meeting (Chicago, IL) in June 2022, the American Society of Clinical Oncology Quality Care Symposium (Chicago, IL) in September 2022, and the San Antonio Breast Cancer Symposium (San Antonio, TX) in December 2022.

## Supporting information


Data S1.

## Data Availability

Data are available from Translational Breast Cancer Research Consortium on reasonable request.
