# Supplementary material for: TBCRC 057: Survey about willingness to participate in cancer clinical trials during the pandemic
Source: Cancer Med. 2024 Mar 11;13(5):e7090. doi: 10.1002/cam4.7090 (PMC10926883; doi:10.1002/cam4.7090)
Supplement: Supplementary file 1 — Data S1. [file CAM4-13-e7090-s001.docx]

**Supplemental Table 1 (Online Only): Clinical Trials Knowledge Assessment**

| **Knowledge True/False Assessment Question** | **Percentage of respondents who responded correctly**  **N=385** |
| --- | --- |
| In a randomized clinical trial, the treatment you get is decided by chance  *Correct response: True* | 68.1% |
| Clinical trials are only used when standard treatments have not worked  *Correct response: False* | 72.2% |
| Clinical trials test treatments which nobody knows anything about  *Correct response: False* | 75.8% |
| Randomized clinical trials are the best way to find out whether one treatment is better than another  *Correct response: True* | 58.4% |
| Clinical trials are not appropriate for serious diseases like cancer  *Correct response: False* | 89.4% |
| My doctor would know which treatment in a clinical trial was better  *Correct response: False* | 43.9% |
| My doctor would make sure I got the better treatment in a clinical trial  *Correct response: False* | 56.1% |
| My previous treatment and overall health will influence my ability to participate in a clinical trial  *Correct response: True* | 76.4% |
| The law requires that doctors and nurses explain all procedures and risks and benefits when offering a clinical trial to a participant  *Correct response: True* | 86.2% |
| Clinical trials have contributed to advances in MBC treatment  *Correct response: True* | 88.8% |
| Participants in clinical trials will have their medical records and names published  *Correct response: False* | 79.0% |

Abbreviation: MBC=metastatic breast cancer

**Supplemental Table 2 (Online Only): Clinical Trials Experience**

|  | | N=385 |
| --- | --- | --- |
| Has a breast cancer provider ever discussed a breast cancer clinical trial with you? – N (%) | Yes | 157 (40.8) |
|  | No | 198 (51.4) |
|  | Missing | 30 (7.8) |
| Has a breast cancer provider discussed a breast cancer clinical trial with you during the COVID-19 pandemic? – N (%) | Yes | 88 (22.9) |
|  | No | 267 (69.4) |
|  | Missing | 30 (7.8) |
| Have you ever participated in a breast cancer clinical trial? – N (%) | Yes | 71 (18.4) |
|  | No | 284 (73.8) |
|  | Missing | 30 (7.8) |
| Are you currently participating in a breast cancer clinical trial?^a^ – N (%) | Yes | 37 (9.6) |
|  | No | 318 (82.6) |
|  | Missing | 30 (7.8) |

a. Of the 37 current trial participants, 11 (29.7%) began participation prior to the onset of the pandemic and 26 (70.3%) began participation during the pandemic.

**Supplemental Table 3 (Online Only): Association of Reluctance to Participate Due to Fear of SARS-CoV-2 Exposure with Reactions Toward Virtual Trial Activities**

| **Virtual Trial Activity** | | **Selected Fear of SARS-CoV-2 Exposure as a Reason for Reluctance^a^**  **N=51** | **Did not Select Fear of SARS-CoV-2 Exposure as a Reason for Reluctance^a^**  **N=134** | **p-value** |
| --- | --- | --- | --- | --- |
| Telemedicine visits with study doctor | | N=48 | N=123 |  |
|  | Much or somewhat more likely – N (%) | 35 (72.9) | 63 (51.2) | 0.01 |
|  | Would not affect my decision whether or not to participate, somewhat less likely, much less likely – N (%) | 13 (27.1) | 60 (48.8) |  |
| Online consent | | N=48 | N=122 |  |
|  | Much or somewhat more likely – N (%) | 36 (75.0) | 57 (46.7) | 0.001 |
|  | Would not affect my decision whether or not to participate, somewhat less likely, much less likely – N (%) | 12 (25.0) | 65 (53.3) |  |
| Online study questionnaires | | N=48 | N=124 |  |
|  | Much or somewhat more likely – N (%) | 37 (77.1) | 61 (49.2) | 0.001 |
|  | Would not affect my decision whether or not to participate, somewhat less likely, much less likely – N (%) | 11 (22.9) | 63 (50.8) |  |

a. Respondents who were not current trial participants and who indicated anything other than being “definitely willing” to participate in a trial during the pandemic were considered reluctant to participate during the pandemic.

**Supplemental Figure 1 (Online Only).** Distribution of Pandemic-Related Anxiety Scores.


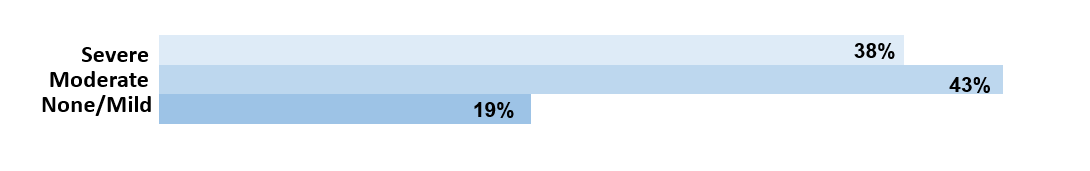


Figure displays the percentages of respondents with mild, moderate and severe pandemic-related anxiety. Pandemic-related anxiety was rated on an 11-point scale (0-no anxiety to 10-worst anxiety possible). Scores of 0-3 were considered none/mild, scores of 4-6 were considered moderate and scores of 7-10 were considered severe. Pandemic-related anxiety was missing for 11 respondents and percentages represent the proportions of the 374 respondents for whom pandemic-related anxiety was available.

## APPENDIX E: Survey

*Note: At the beginning of the survey, respondents will be offered the choice to select their preferred language: English or Spanish.*

*Note: Wording in the introduction and consent section regarding whether the survey is IRB approved or exempt will be modified once this has been determined.*

*INTRODUCTION AND CONSENT:*

You are being asked to complete an online research survey from the Translational Breast Cancer Research Consortium. We are conducting this research because we want to learn whether the COVID-19 pandemic affects how people feel about participating in breast cancer clinical trials. Breast cancer clinical trials are a type of research done to learn about breast cancer and about how to improve breast cancer treatments.

Your responses to this survey will build on prior research about barriers to clinical trial participation and may help breast cancer researchers improve how they conduct breast cancer clinical trials during and after the pandemic.

Adults diagnosed with breast cancer who live in the United States are eligible to participate in this research survey if they…

…Have metastatic breast cancer

OR

…Were diagnosed with breast cancer of any stage within the past 5 years

This survey was *approved by/deemed exempt by* the Johns Hopkins School of Medicine Institutional Review Board. Completing this survey is optional and your answers will be kept anonymous. By beginning this survey, you are providing consent to participate in this research.

This survey will take approximately 15 minutes of your time. Please mark only one answer for each question unless otherwise indicated. Please do not complete this survey more than once.

If you would like more information about this survey, please contact Dr. Karen Lisa Smith by email at [ksmith60@jhmi.edu](mailto:ksmith60@jhmi.edu) or by phone at (202) 660-6500.

If you know other people who you think may be interested in this survey, please forward the link to them.

Clinic HERE to begin the survey.

*FIRST, PLEASE TELL US ABOUT YOURSELF SO WE CAN MAKE SURE YOU ARE ELIGIBLE TO PARTICIPATE IN THIS RESEARCH SURVEY:*

1. Have you ever been diagnosed with breast cancer?

- Yes
- No

*Branching logic:*

- *If “Yes”: Continue to question 2*
- *If “No”: We appreciate your interest, but this survey is for people who have been diagnosed with breast cancer. Thank you for your time.*

2. How many years ago were you first diagnosed with breast cancer?

- Less than 1 year ago
- 1-5 years ago
- 5-10 years ago
- More than 10 years ago

3. Which of the following statements most accurately describes you?

- I have been diagnosed with early stage breast cancer (breast cancer involving the breast with or without involving the armpit lymph glands)
- I have been diagnosed with metastatic breast cancer (breast cancer that has spread to other parts of the body)
- Other

*Branching logic:*

- *If respondent has indicated at least one of the following responses to questions 2 and 3, proceed to question 4:*
  - *Response to question 2 is “Less than 1 year ago”*
  - *Response to question 2 is “1-5 years ago”*
  - *Response to question 3 is “I have been diagnosed with metastatic breast cancer (breast cancer that has spread to other parts of the body)”*
- *If none of the above responses are indicated for questions 2 and 3: We appreciate your interest, but this survey is for people with metastatic breast cancer or who were diagnosed with breast cancer of any stage within the past 5 years. Thank you for your time.*

4. How old are you today? __________________ (enter a number of years)

*Branching logic:*

- *If* ≥18*: Continue question 5*
- *If “<18”: We appreciate your interest, but this survey for people who are age 18 years or older. Thank you for your time.*

5. Where do you live?

- United States
- Another country besides United States

*Branching logic:*

- *If “United States”: Continue to question 6*
- *If “Another country besides United States”: We appreciate your interest, but this survey is for people who live in the United States. Thank you for your time.*

*TELL US ABOUT YOUR EXPERIENCE DURING THE COVID-19 PANDEMIC:*

6. Have you ever been near someone that you know, or suspect, had COVID-19 at the time (such as co-workers, family members, or others)? Please select all that apply.

- Yes, known COVID-19
- Yes, suspected COVID-19
- Not that I know of

7. Have you ever been tested for COVID-19?

- Yes – more than once
- Yes – once
- No

*Branching logic:*

- *If “Yes-more than once” or “Yes – once”: Continue to question 8*

*If “No”: Go to question 9*

8. Have you ever had a positive test for COVID-19?

- Yes
- No
- Waiting for results

9. Do you think you have had COVID-19?

- Yes
- No
- Maybe

10. Some people are anxious about the COVID-19 pandemic. Using the scale below, please mark the number that corresponds to how anxious you feel about the COVID-19 pandemic.

0 1 2 3 4 5 6 7 8 9 10

No anxiety………………………………………………………Worst anxiety possible

*Branching logic:*

- *If “≥7”: Please contact your health care provider if you are bothered by anxiety. Then, continue to question 11*

*If “<7”: Go directly to question 11*

11. Have you had a telemedicine appointment (via internet-based video or telephone connection) with a breast cancer provider during the COVID-19 pandemic?

- Yes
- No

12. Have you had surgery for breast cancer within the past 6 weeks?

- Yes
- No

13. Have you had radiation for breast cancer within the past 6 weeks?

- Yes
- No

14. Have you received chemotherapy or any other injectable medication for breast cancer within the past 6 weeks?

- Yes
- No

15. Are you taking any medications by mouth to treat breast cancer or to prevent breast cancer from returning?

- Yes
- No

16. Have you received at least one dose of a COVID-19 vaccine?

- Yes
- No

*Branching logic:*

- *If* “*Yes”*: *Go to question 19*

*If “No”: Continue to question 17*

17. Once it becomes available to you, how likely are you get a COVID-19 vaccine?

- Extremely likely
- Somewhat likely
- Unsure
- Somewhat unlikely
- Extremely unlikely

*Branching logic:*

- *If “Extremely likely” or “Somewhat likely”: Go to question 19*

*If “Unsure”, “Somewhat unlikely” or “Extremely unlikely”: Continue to question 18*

18. What is your primary reason for feeling you may be unlikely or unsure about getting a COVID-19 vaccine?

- I may not be able to afford it
- I do not have a clinic or provider where I will be able to get the vaccine
- I have a health condition that may limit my ability to receive the vaccine
- I have concerns about the safety of the vaccine
- Other

*THE NEXT SET OF QUESTIONS ARE ABOUT CLINICAL TRIALS:*

Many people have not had any experience with clinical trials before. Please indicate whether you think the following statements about clinical trials are true or not.

19. In a randomized clinical trial, the treatment you get is decided by chance

- True
- False
- Don’t know

20. Clinical trials are only used when standard treatments have not worked

- True
- False
- Don’t know

21. Clinical trials test treatments which nobody knows anything about

- True
- False
- Don’t know

22. Randomized clinical trials are the best way to find out whether one treatment is better than another

- True
- False
- Don’t know

23. Clinical trials are not appropriate for serious diseases like cancer

- True
- False
- Don’t know

24. My doctor would know which treatment in a clinical trial was better

- True
- False
- Don’t know

25. My doctor would make sure I got the better treatment in a clinical trial

- True
- False
- Don’t know

26. My previous treatment and overall health will influence my ability to participate in a clinical trial

- True
- False
- Don’t know

27. The law requires that doctors and nurses explain all procedures and risks and benefits when offering a clinical trial to a participant

- True
- False
- Don’t know

28. Clinical trials have contributed to advances in metastatic breast cancer treatment

- True
- False
- Don’t know

29. Participants in clinical trials will have their medical records and names published

- True
- False
- Don’t know

Please tell us about your experience with clinical trials:

30. Has a breast cancer provider ever discussed a breast cancer clinical trial with you?

- Yes
- No

*Branching logic:*

- *If “Yes”: Continue to question 31*
- *If “No”: Go to question 32*

31. Has a breast cancer provider discussed a breast cancer clinical trial with you during the COVID-19 pandemic?

- Yes
- No

32. Have you ever participated in a breast cancer clinical trial?

- Yes
- No

*Branching logic:*

- *If “Yes”: Continue to question 33*
- *If “No”: Go to question 35*

33. Are you currently participating in a breast cancer clinical trial?

- Yes
- No

*Branching logic:*

- *If “Yes”: Continue to question 34*
- *If “No”” Go to question 36*

34. When did you begin participating in the breast cancer clinical trial?

- Before the COVID-19 pandemic started
- During the COVID-19 pandemic

*Branching logic:*

- *If “Before the COVID-19 pandemic started”: Go to next section for question 38*
- *If “During the COVID-19 pandemic”: Continue to question 35*

35. Please mark the number that indicates how willing you would have been to consider participating in a breast cancer clinical trial before the COVID-19 pandemic. If you were diagnosed with breast cancer during the pandemic, please mark the number that indicates how willing you think you would have been to consider participating in a breast cancer clinical trial if you had been diagnosed before the pandemic.

0 1 2 3 4

Not at all willing………………………………………Definitely willing

*Branching logic:*

- *If response to question 34 was “During the COVID-19 pandemic”: Go to next section for question 38*
- *If 34 was not answered due to branching logic: Continue to question 36*

36. Please mark the number that indicates how willing you would be to consider participating in a breast cancer clinical trial during the COVID-19 pandemic.

0 1 2 3 4

Not at all willing………………………………………Definitely willing

*Branching logic:*

- *If response is “4”, skip to next section for question 38*
- *If response is 0-3, continue to question 37*

37. Please tell us if any of the following are reasons why you would not definitely be willing to consider participating in a breast cancer clinical trial during the COVID-19 pandemic. Mark all that apply:

- Fear of exposure to SARS-CoV-2 (the coronavirus that causes COVID-19)
- Do not want to spend time away from home/family
- Financial concerns
- Health insurance concerns
- Cannot get time off work
- Difficulty with transportation to see health care providers
- Don’t feel safe
- Worry about side effects
- Don’t have time
- Too much trouble
- Worry about delaying cancer treatment
- Worry about not getting the best cancer treatment
- Prefer to choose my own cancer treatment
- My family would not support my participation in a clinical trial
- Don’t trust doctors and nurses doing cancer research
- Not interested in clinical trials
- Too overwhelmed

People who participate in breast cancer clinical trials often have to attend appointments and have to undergo treatments, x-ray tests, biopsy procedures and laboratory tests, many of which are done at the cancer clinic conducting the clinical trial. Some of the tests affect breast cancer care while others are done for research purposes only.

During the COVID-19 pandemic, researchers have cut back or changed requirements for people participating in clinical trials. Some researchers think these changes should continue after the pandemic also.

Please tell us whether the following changes would affect your decision to participate in a clinical trial during or after the COVID-19 pandemic. If you are currently participating in a clinical trial, please indicate whether the following changes would affect your decision to participate in another clinical trial during or after the COVID-19 pandemic:

| **CHANGE LOCATION:** If you could complete the following trial requirements at a **location close to your home** **instead of traveling to the clinical trial site** to do them, how would this affect your decision to participate? | | | | | |
| --- | --- | --- | --- | --- | --- |
|  | Much less likely to participate | Somewhat less likely to participate | Would not affect my decision whether or not to participate | Somewhat more likely to participate | Much more likely to participate |
| 38. Blood tests | □ | □ | □ | □ | □ |
| 39. X-ray tests | □ | □ | □ | □ | □ |
| 40. Doctor visits (i.e. you would see a doctor close to home who is not involved in the clinical trial instead of the clinical trial doctor) | □ | □ | □ | □ | □ |

| **SWITCH CLINICAL TRIAL PROCEDURES TO SECURE TELEMEDICINE PORTAL:** If you could complete the following trial requirements **electronically instead of traveling to the clinical trial site to do** **them in person**, how would this affect your decision to participate? | | | | | |
| --- | --- | --- | --- | --- | --- |
|  | Much less likely to participate | Somewhat less likely to participate | Would not affect my decision whether or not to participate | Somewhat more likely to participate | Much more likely to participate |
| 41. Doctor visits (i.e. you would see the clinical trial doctor via telemedicine using phone or video connection) | □ | □ | □ | □ | □ |
| 42. Sign online forms to consent to participate in the trial | □ | □ | □ | □ | □ |
| 43. Complete online questionnaires about how you are feeling | □ | □ | □ | □ | □ |

| **MAKING TRIAL SCHEDULE FLEXIBLE AND CONVENIENT**: If the following aspects of the clinical trial were flexible and convenient, how would it affect your decision to participate? | | | | | |
| --- | --- | --- | --- | --- | --- |
|  | Much less likely to participate | Somewhat less likely to participate | Would not affect my decision whether or not to participate | Somewhat more likely to participate | Much more likely to participate |
| 44. I would not need to come to the clinical trial site more frequently than once every 3 weeks. | □ | □ | □ | □ | □ |
| 45. I could opt out of blood tests for the clinical trial that are for research only (i.e. that do not affect my care) | □ | □ | □ | □ | □ |
| 46. I could opt out of biopsies for the clinical trial that are for research only (i.e. that do not affect my care) | □ | □ | □ | □ | □ |
| 47. Rather than completing doctor visits, treatments, tests or procedures for the clinical trial on a specific day, I could have the option of completing them a few days earlier or a few days later | □ | □ | □ | □ | □ |
| 48. Rather than coming to the clinical trial site to pick up medications taken by mouth for the clinical trial, I could have the medications delivered to my home. | □ | □ | □ | □ | □ |

For the next questions, please mark the number indicating how much you agree or disagree with each of the statements about clinical trials:

|  | Strongly Disagree Strongly Agree | | | | | | |
| --- | --- | --- | --- | --- | --- | --- | --- |
| 49. I’d get improved cancer treatment if I took part in a clinical trial | 1  □ | 2  □ | 3  □ | 4  □ | 5  □ | 6  □ | 7  □ |
| 50. People who join clinical trials have a better chance of beating their cancer | 1  □ | 2  □ | 3  □ | 4  □ | 5  □ | 6  □ | 7  □ |
| 51. Joining a clinical trial would mean I’d receive the best existing cancer treatment | 1  □ | 2  □ | 3  □ | 4  □ | 5  □ | 6  □ | 7  □ |
| 52. By joining a clinical trial, I would receive better health care | 1  □ | 2  □ | 3  □ | 4  □ | 5  □ | 6  □ | 7  □ |
| 53. Taking part in a clinical trial is a lot more trouble than just getting the usual treatment | 1  □ | 2  □ | 3  □ | 4  □ | 5  □ | 6  □ | 7  □ |
| 54. Getting treatment in a clinical trial is less safe than getting the usual cancer treatment | 1  □ | 2  □ | 3  □ | 4  □ | 5  □ | 6  □ | 7  □ |
| 55. Treatments received in a clinical trial could be unsafe for myself | 1  □ | 2  □ | 3  □ | 4  □ | 5  □ | 6  □ | 7  □ |
| 56. My taking part in a clinical trial could lead to more health problems | 1  □ | 2  □ | 3  □ | 4  □ | 5  □ | 6  □ | 7  □ |
| 57. Joining a clinical trial would make cancer treatment more difficult | 1  □ | 2  □ | 3  □ | 4  □ | 5  □ | 6  □ | 7  □ |
| 58. In general, people should know more about clinical trials | 1  □ | 2  □ | 3  □ | 4  □ | 5  □ | 6  □ | 7  □ |
| 59. Clinical trials are of little importance to me | 1  □ | 2  □ | 3  □ | 4  □ | 5  □ | 6  □ | 7  □ |
| 60. Access to cancer treatment clinical trials is important to me | 1  □ | 2  □ | 3  □ | 4  □ | 5  □ | 6  □ | 7  □ |
| 61. People who take part in clinical trials are helping all of us fight cancer | 1  □ | 2  □ | 3  □ | 4  □ | 5  □ | 6  □ | 7  □ |
| 62. I feel certain my safety would be watched closely in a clinical trial | 1  □ | 2  □ | 3  □ | 4  □ | 5  □ | 6  □ | 7  □ |
| 63. Doctors and nurses tell patients the truth about what to expect during a clinical trial | 1  □ | 2  □ | 3  □ | 4  □ | 5  □ | 6  □ | 7  □ |
| 64. If I took part in a clinical, I would be treated like a guinea pig | 1  □ | 2  □ | 3  □ | 4  □ | 5  □ | 6  □ | 7  □ |
| 65. Doctors and nurses mislead their patients who are involved in clinical trials | 1  □ | 2  □ | 3  □ | 4  □ | 5  □ | 6  □ | 7  □ |
| 66. It would be safe for me to join a clinical trial for treatment | 1  □ | 2  □ | 3  □ | 4  □ | 5  □ | 6  □ | 7  □ |
| 67. I will get my needed treatment as soon as possible if I am in a clinical trial | 1  □ | 2  □ | 3  □ | 4  □ | 5  □ | 6  □ | 7  □ |
| 68. If I am in a clinical trial I will have to spend extra time having more tests and doctor visits | 1  □ | 2  □ | 3  □ | 4  □ | 5  □ | 6  □ | 7  □ |
| 69. I may have to spend more time and money on transportation and childcare and may lose income due to time away from work if I participate in a clinical trial | 1  □ | 2  □ | 3  □ | 4  □ | 5  □ | 6  □ | 7  □ |

*FINALLY, PLEASE TELL US MORE ABOUT YOURSELF:*

70. What state do you live in? (Drop down menu of states)

71. What county do you live in? (Drop down menu of counties based on state respondent lives in)

72. What is your ZIP code? ________________________ (enter a 5-digit number)

73. Please indicate your gender identity:

- Cisgender Female (assigned female at birth and identify as female)
- Cisgender Male (assigned male at birth and identify as male)
- Transgender Female (assigned male at birth and identify as female)
- Transgender Male (assigned female at birth and identify as male)
- Genderqueer or Non-Binary (neither exclusively male nor female)
- Another gender category
- I choose not to disclose

74. What is your race? (Check all that apply)

- American Indian/Alaska Native
- Asian
- Black/African American
- Native Hawaiian or Other Pacific Islander
- White/Caucasian
- Other

75. Are you of Hispanic or Latino(a) origin or descent?

- Yes
- No

76. Please indicate your highest level of academic achievement:

- Less than a high school diploma or equivalent
- High school diploma or equivalent
- Some college, no degree
- Associate’s degree
- Bachelor’s degree
- Master’s or doctoral degree

77. Please choose the option that best describes your current marital status.

- Single
- Married/Living with partner
- Partnered/Not living together
- Widowed
- Divorced
- Separated
- Other

78. Please indicate your current household income per year in US dollars.

- Less than $20,000
- $20,000 to $34,999
- $35,000 to $49,999
- $50,000 to $74,999
- $75,000 to $99,999
- More than $100,000

79. What is your current employment status? Please select what you consider to be your main activity.

- Working full time (≥32 hours/week)
- Working part time (1-31 hours/week)
- Unemployed, looking for work
- Unemployed, not looking for work
- In job training
- Temporarily laid off (no pay)
- Retired
- On short term disability
- On long term disability
- Permanently disabled
- I do not work
- In school
- Other

*Branching logic:*

- *If response is “Working full time (≥32 hours/week)” or “Working part time (1-31 hours per week)”: Continue to question 80*
- *All other responses: Go to question 81*

80. Please indicate the primary location of your work during the pandemic

- I work from my home
- I leave my home to go to work

81. Please indicate your health insurance type. Choose all that apply.

- Private (e.g. BlueCross, Cigna)
- Medicare
- Medicaid
- Tri-Care/Other Military
- Indian Health Service
- The Veterans Health Administration (VA)
- None
- Other

82. Do you have school-aged children (kindergarten through 12^th^ grade) living with you at home?

- Yes
- No

83. How old were you when you were first diagnosed with breast cancer?

- Under 18
- 18-29
- 30-39
- 40-49
- 50-59
- 60-69
- 70 or older

84. How long does it take for you to travel to an appointment to see your breast cancer provider? If you have more than one breast cancer provider, please indicate the time it takes for you to travel to an appointment with the provider you see the most frequently.

- Less than 15 minutes
- 15-30 minutes
- 30-60 minutes
- 60-90 minutes
- More than 90 minutes

85. Which of the following best describes where you primarily receive your breast cancer treatment or follow-up care?

- Academic medical center
- Community based/private practice
- Not sure

­­­­­­­­­­­­­­­­­­­­­­­­­-------------------------------------------------------------------------------------------------------------------------------

Thank you for completing this survey. This research is supported by an award from the Metastatic Breast Cancer Network.  The Translational Breast Cancer Research Consortium is grateful for its support from Susan G. Komen and the Breast Cancer Research Foundation.

Click here to submit your survey.

-----------------------------------------------------------------------------------------------------------------------------
